# Environmental dependency of ectomycorrhizal fungi as soil organic matter oxidizers

**DOI:** 10.1111/nph.20205

**Published:** 2024-10-17

**Authors:** Qiuyu Chen, Ilya Strashnov, Bart van Dongen, David Johnson, Filipa Cox

**Affiliations:** ^1^ Department of Earth and Environmental Sciences The University of Manchester Manchester M13 9PT UK

**Keywords:** *Amanita rubescens*, ectomycorrhizal fungi, *Hebeloma velutipes*, inorganic nitrogen availability, interspecific interactions, *Lactarius rufus*, soil organic matter, *Suillus variegatus*

## Abstract

- Forest soils play a pivotal role as global carbon (C) sinks, where the dynamics of soil organic matter (SOM) are significantly influenced by ectomycorrhizal (ECM) fungi. While correlations between ECM fungal community composition and soil C storage have been documented, the underlying mechanisms behind this remain unclear.
- Here, we conducted controlled experiments using pure cultures growing on naturally complex SOM extracts to test how ECM fungi regulate soil C and nitrogen (N) dynamics in response to varying inorganic N availability, in both monoculture and mixed culture conditions.
- ECM species dominant in N‐poor soils exhibited superior SOM decay capabilities compared with those prevalent in N‐rich soils. Inorganic N addition alleviated N limitation for ECM species but exacerbated their C limitation, reflected by reduced N compound decomposition and increased C compound decomposition. In mixed cultures without inorganic N supplementation, ECM species with greater SOM decomposition potential facilitated the persistence of less proficient SOM decomposers. Regardless of inorganic N availability, ECM species in mixed cultures demonstrated a preference for C over N, intensifying relatively labile C compound decomposition.
- This study highlights the complex interactions between ECM species, their nutritional requirements, the nutritional environment of their habitat, and their role in modifying SOM.

## Introduction

Forests constitute a significant reservoir of carbon (C), the majority of which is stored belowground, primarily in the form of soil organic matter (SOM) (Pan *et al*., 2011; Schmidt *et al*., 2011). The decomposition of SOM in forests is integral to the global cycling of C and nitrogen (N), underpinning diverse and critical forest ecosystem services such as climate regulation, biomass production and habitat provision for forest species (Deluca & Boisvenue, 2012). Within temperate and boreal forests, evidence increasingly suggests that ectomycorrhizal (ECM) fungi are involved in the decomposition of SOM (Phillips *et al*., 2014; Lindahl *et al*., 2021) mainly to capture and immobilize N into their tissues, which they can then exchange with their plant hosts for photosynthetically derived C (Lindahl & Tunlid, 2015; Baldrian, 2017). However, our understanding of how SOM decomposition differs across ECM fungal species and environmental contexts is in its infancy. These fundamental gaps pose challenges to the refinement of strategies aimed at optimizing C sequestration within the context of climate change.

ECM fungi originate from multiple phylogenetic groups and their ability to decompose SOM exhibits considerable variation across evolutionary lineages (Kohler *et al*., 2015; Pellitier & Zak, 2018). For example, *Amanita muscaria*, which evolved within a clade of brown rot saprotrophs, has undergone a genetic loss resulting in a reduced capacity for decomposing SOM (Kohler *et al*., 2015). By contrast, *Hebeloma cylindrosporum*, descended from a white‐rot ancestor that used class II fungal peroxidases to oxidize SOM, has retained three manganese peroxidase genes for SOM decomposition (Kohler *et al*., 2015). Furthermore, the genome of *Cortinarius glaucopus* contains 11 peroxidases, a number comparable to that observed in numerous white‐rot wood decomposers, underscoring their likely significant contribution to the decomposition of SOM within forest ecosystems (Bödeker *et al*., 2009; Miyauchi *et al*., 2020). Given the inherent functional heterogeneity of ECM fungi, shifts in their community composition are likely to drive distinct and profound effects on C and N cycling within forest ecosystems (Sterkenburg *et al*., 2018; Lindahl *et al*., 2021).

An important driver of ECM fungal community composition is the availability of inorganic N (Zak *et al*., 2019), which can also act as a regulator of ECM‐mediated SOM decomposition (Bogar *et al*., 2021; Argiroff *et al*., 2022). Recent findings demonstrated that ECM fungal communities thriving in environments characterized by limited inorganic N content manifest an elevated genomic capacity for SOM decomposition (Mayer *et al*., 2023). These communities are often characterized by the prevalence of genera such as *Cortinarius* and *Hebeloma* (Pellitier & Zak, 2021). By contrast, ECM communities in soils with high inorganic N concentrations are typically dominated by genera such as *Scleroderma* and *Russula*, which have a weaker capacity for SOM decay (van der Linde *et al*., 2018). Other studies have revealed significant positive correlations between lignin‐derived SOM and soil C content with inorganic N availability (Argiroff *et al*., 2022). This association is attributed to the presence of ECM fungi equipped with peroxidase enzymes, which exhibit diminished occurrence with increasing inorganic N availability (Clemmensen *et al*., 2015; Argiroff *et al*., 2022). Interactions between soil N availability, ECM fungal community composition, and soil C sequestration have been demonstrated within natural forest ecosystems, but the intricate mechanisms underpinning these relationships remain unresolved.

Along with variations in soil chemistry, interspecific interactions among ECM fungi exert significant influence on the structure of entire ECM communities, consequently impacting SOM dynamics (Kennedy, 2010; Fernandez & Kennedy, 2016). Studies have demonstrated that competition for N resources between ECM fungi and free‐living decomposers can slowdown overall soil C cycling and increase soil C storage (Averill & Hawkes, 2016; Fernandez *et al*., 2020). However, how interactions between ECM fungal species might also alter rates of C cycling remains unclear, even though interspecific competition for resources within this group has been widely demonstrated (Koide *et al*., 2005; Kennedy, 2010; Smith *et al*., 2023) and is recognized as a key determinant shaping their community composition (Kennedy, 2010) and structure (Pickles *et al*., 2012). Interspecific interactions between ECM species may lead to similar inhibition, or alternatively could enable facilitation, whereby those species possessing more powerful decomposition strategies free up nutrients from recalcitrant soil compounds, enabling poorer decomposers to persist, in turn accelerating soil C cycling (Tiunov & Scheu, 2005; Lindahl & Tunlid, 2015). The lack of studies employing natural composite SOM extracts in competition experiments involving ECM fungi contributes to the uncertainty surrounding the impact of species interactions on SOM decomposition.

Here, we conducted controlled pure culture experiments to address the knowledge gaps surrounding the mechanisms and context‐dependency of SOM decomposition by ECM fungi, strengthening our understanding of C and N cycling in forest ecosystems. We identified and cultured ECM species that thrived under conditions of low inorganic N availability and that were expected to demonstrate enhanced capacity to decompose SOM, alongside ECM species typically from soils with high inorganic N availability. We used these isolates to explore the context‐dependency of SOM decomposition, testing the hypothesis that an increase in inorganic N availability would lead to reduced ECM fungal decomposition of N compounds, while potentially enhancing their decomposition of C compounds as a regulatory mechanism to offset the C and N imbalance induced by inorganic N supplementation. Additionally, we tested the hypothesis that interspecific interactions might negatively affect ECM fungal growth, with the magnitude of this impact varying by fungal identity and environmental contexts, consequently shaping SOM decomposition patterns. Analyses of fungal growth and extracellular enzyme production were paired with pyrolysis gas chromatography mass spectrometry (Py‐GC‐MS) to examine the SOM dynamics at the molecular level.

## Materials and Methods

### Fungal species

Four ECM species, namely *Hebeloma velutipes* Bruchet, *Suillus variegatus* (Sw. Fr.) O. Kuntze, *Amanita rubescens* (Pers. Fr) SF Gray., and *Lactarius rufus* (Scop. Fr.) Fr., were selected in this study. Three of the isolates (i.e. *Hebeloma velutipes*, *Suillus variegatus*, and *Lactarius rufus*) were sourced from an 88‐year‐old *Pinus sylvestris* forest in Sweden (64°07′N, 17°33′E), where replicate plots have been fertilized for several decades (Jacobson & Pettersson, 2010). *Lactarius rufus* was isolated from a fruit body occurring within a plot that has received N fertilization for 39 yr at a rate of 75 kg N ha^−1^ yr^−1^, and *Hebeloma velutipes* and *Suillus variegatus* were from fruit bodies in adjacent nonfertilized control plots. The selection of *Amanita rubescens* was based on its typical dominance in N‐rich sites (Wolfe *et al*., 2012). The observed differences in these species abundance across varying levels of inorganic N, alongside the significant variation in the potential for secreted carbohydrate‐active enzymes (CAZymes) among these fungal taxa reported by Miyauchi *et al*. (2020), suggest differences in the capacity of these species for SOM decomposition and their responsiveness to environmental contexts.

### Culture conditions

Cultures of the four ECM species were grown in Petri dishes on a layer of glass beads immersed in the liquid minimum Melin‐Norkrans (MMN) medium (composition: 10 g l^−1^ glucose, 0.5 g l^−1^ KH_2_PO_4_, 0.25 g l^−1^ (NH_4_)_2_HPO_4_, 0.15 g l^−1^ MgSO_4_·7H_2_O, 0.025 g l^−1^ NaCl, 0.05 g l^−1^ CaCl_2_·2H_2_O, 0.012 g l^−1^ FeCl_3_·6H_2_O, and 3 g l^−1^ Malt Extract; pH 4.0) (Rineau *et al*., 2012). A monolayer of autoclaved 4 mm diameter glass beads was poured into the bottom of a 9 cm Petri dish, and 10 ml of MMN medium was added. Two mycelial plugs (each *c*. 5 mm in diameter), sourced from the growing margin of an active mycelium belonging to either the same or distinct ECM species, were subsequently transferred to the glass bead plates. Given the variance in growth rates among isolates of each species, the incubation duration was adjusted based on the mycelium diameter, ensuring that the size of each clump was *c*. 2 cm. As a result, *Hebeloma velutipes* and *Suillus variegatus* underwent incubation for *c*. 15 d, *Amanita rubescens* for *c*. 30 d, and *Lactarius rufus* for *c*. 60 d. In mixed cultures, the species requiring a longer growth period was inoculated first, followed by the other species after a specified interval. Following the respective incubation periods, the medium was replaced with N‐free MMN medium to induce the formation of an N‐deprived mycelium. After 24 h, the mycelium was washed with sterile Milli‐Q water, and finally, SOM extracts (10 ml) were added. Cultures were then incubated in SOM extracts for 30 d at 22°C in the dark for the decomposition study. After 30 d of incubation, all mycelium was collected using autoclaved forceps, and SOM extracts were subsequently obtained with a sterile syringe fitted with a fine needle (Supporting Information Fig. S1). Decomposition of SOM by ECM species was examined in the absence/presence of an inorganic N source (ammonium supplied as (NH_4_)_2_HPO_4_, 0.2 g l^−1^), and in both monoculture and mixed culture conditions. Controls, with no ECM species incorporation, were set up replicating the same steps. Each treatment had 4 replicates, for a total of 88 samples in this study.

### Preparation of SOM extracts

Soil was collected from the upper 10 cm layer of nonfertilized control plots within the same *Pinus sylvestris* forest described above. SOM was extracted from the soil using hot water (Davidson *et al*., 1987). In brief, 120 g of field‐moist unsieved soil was combined with 600 ml Milli‐Q water and boiled for 1 h, and SOM extracts were vacuum‐filtered through a 0.2‐μm Stericup system (Millipore) to remove particles and sterilize the extracts (Rineau *et al*., 2012). The final volume of SOM extracts was adjusted to 400 ml, and extracts were supplemented with glucose to a final concentration similar to that in the MMN medium (glucose of 10 g l^−1^). Notably, Py‐GC‐MS revealed the presence of major biomolecule classes typical of forest soils in the SOM extracts (Table S1). Additionally, significant positive correlations were observed between the properties of forest soil and those of SOM extracts (Table S2), confirming that SOM extracts accurately represent forest soils (Shah *et al*., 2016).

### Analysis of fungal biomass and enzyme activities

The mycelium was gathered on Whatman glass filter paper and dried in an oven at 70°C for 24 h, and the dry weight was recorded as ECM fungal biomass.

The enzymatic activities of β‐glucosidase (GLC), β‐xylosidase (XYL), phenol oxidase (POX), and peroxidase (PER) within SOM extracts were measured according to previous studies (Rineau *et al*., 2012; Cordero *et al*., 2023) with appropriate adjustments to our samples. In brief, GLC and XYL were assessed using *p*NP‐linked substrate analogues (Jackson *et al*., 2013), and POX and PER were determined through the oxidation of l‐DOPA (Sinsabaugh & Linkins, 1988). To minimize the influence of preexisting enzymes in SOM extracts, we used SOM extracts without ECM species as controls when evaluating these enzyme activities.

### Analysis of SOM extracts

Total organic carbon (TOC) concentrations in SOM extracts were measured using a 5000A TOC analyzer (Shimadzu, Japan). Total nitrogen (TN), ammonia‐N, and nitrate‐N concentrations in SOM extracts were analysed on a Seal AA3 Segmented Flow Multi‐chemistry analyzer (Seal Analytical, Southampton, UK).

Py‐GC‐MS was conducted to analyse the detailed changes in macromolecular composition occurring during decomposition of SOM extracts by ECM species using a CDS‐5200 Pyroprobe (CDS Analytical, Oxford, PA, USA) interfaced to an Agilent GC‐MSD system (Agilent, Santa Clare, CA, USA). Five millilitres of SOM extract was freeze vacuum dried and *c*. 1 mg freeze‐dried sample was placed into a clean fire‐polished quartz tube and pyrolysed at 600°C for 20s in a flow of helium. The released material was transferred via a heated transfer line to an Agilent 7980A GC fitted with a Zebron ZB‐5MS column (Phenomenex, Woerden, the Netherlands; 30 m × 250 μm × 0.25 μm) coupled to Agilent 5975C MSD single quadrupole mass spectrometer in electron ionization mode (scanning a range of *m/z* 50 to 650 at 2.7 scans^−1^; ionization energy: 70 eV) using helium as a carrier gas. The pyrolysis transfer line and rotor oven temperatures were set at 325°C, the heated GC interface at 280°C, the EI source at 230°C and the MS quadrupole at 150°C. The samples were introduced in split mode (split ratio: 20 : 1; constant flow of 2 ml per min, gas saver mode active), and the oven was programmed from 40°C, held for 5 min, rising to 250°C at a rate of 4°C per min before a final rise at 20°C per min to 300°C, which was held for 3 min, resulting in a total run time of 60 min.

Released pyrolysis moieties were identified by comparison of relative retention times and spectra to those reported in the NIST Mass Spectral Library and grouped into a number of groups based on origin and chemical similarity: alkyl compounds, lignin, polysaccharides, phenols, N‐containing compounds, and (poly)aromatics (Table S1). The relative amounts were obtained by comparison of the relative abundance of each pyrolysis moiety with that of all identified moieties and reported as percentage (of total). N‐containing compounds were designated as N compounds, while lignin, and polysaccharides combined were categorized as C compounds, owing to their considerable presence and significant alterations (Fig. S2). Additionally, given the higher resistance of lignin to decomposition compared with polysaccharides (Kögel‐Knabner, 2002), these two classes of compounds were further characterized as relatively recalcitrant and labile C compounds, respectively.

### Statistical analysis

All analyses were performed using R software (v.4.2.2, https://www.r‐project.org/). Principal component analysis (PCA) was employed to visualize compositional shifts in SOM extracts driven by the activity of ECM species. Additionally, the SOM Shannon diversity index was calculated based on the molecular composition of individual SOM extract samples to evaluate SOM decomposition across varied treatments. Independent sample *t*‐test analysis was used to assess the differences in variables between two inorganic N treatments, and one‐way analysis of variance (ANOVA) was used to compare the differences in variables across multiple ECM fungal cultures.

Linear regression analyses were performed to examine the relationships between ECM biomass and the SOM Shannon diversity index, as well as between ECM biomass and N and C compound decomposition. Furthermore, the examination of C compounds was extended to differentiate between relatively recalcitrant and labile forms, represented by lignin and polysaccharides, respectively. Linear regression was employed to clarify the relationships between fluctuations in TOC concentrations and the decomposition of these C compounds. Pearson correlation analyses were used to investigate the associations between the decomposition of these C compounds and variations in enzyme activities.

Structural equation modelling (SEM) was conducted to unravel the underlying context‐dependent mechanisms by which ECM fungi alter soil C and N dynamics. All variables used in SEM were Z‐score transformed to allow comparisons among multiple predictors and models. Adequate model fits were determined according to a nonsignificant chi‐squared test (*P* > 0.05), low AIC value, high comparative fit index (CFI > 0.95) and low standardized root mean squared residual (SRMR < 0.05). The SEMs were conducted using the R package lavaan v.0.5.23.

## Results

### Modifications in SOM extract composition determined by ECM species

**Fig. 1:**
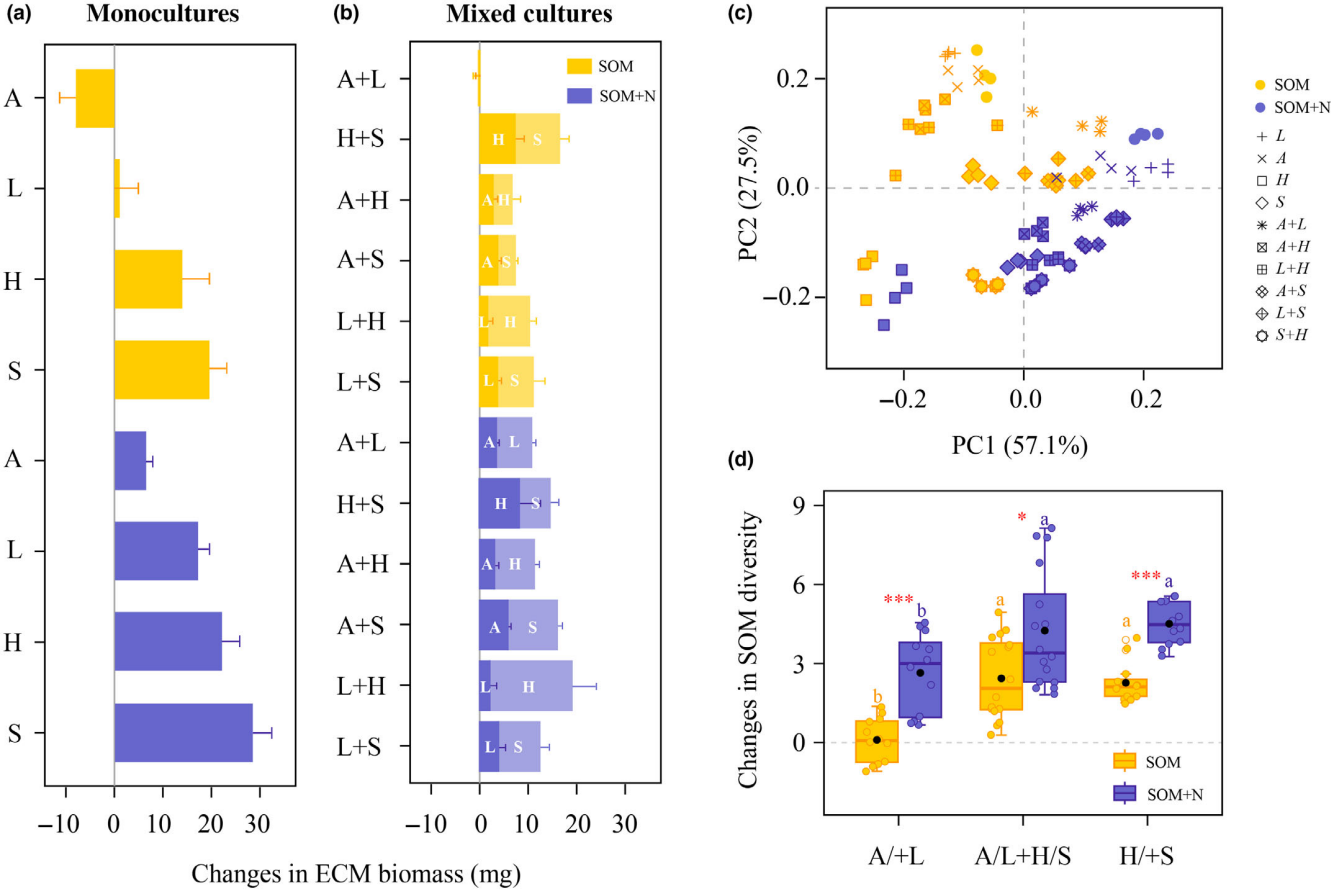
Variations in the capacity of ectomycorrhizal (ECM) species to modify soil organic matter. (a, b) Changes in ECM fungal biomass (mean ± SE; *n* = 4), assessed by comparing mycelial biomass post 30‐d incubation in soil organic matter (SOM) extracts to preincubation levels. (c) Principal component analysis (PCA) depicting the composition of SOM extracts. (d) Changes in the SOM Shannon diversity index, derived from comparisons between measurements in SOM extracts containing ECM species and controls without ECM species, with data from cultures containing ECM species originating from either N‐rich (A, L) or N‐poor (H, S) soils, and mixed cultures comprising species from both soil types. Boxplots indicate medians (lines) and mean values (black points), SE and ranges, and dots are individual data points. Bars sharing a coloured letter are not significantly different (*P* > 0.05); cultures in the absence or presence of supplemental inorganic N analysed independently. Red asterisks represent significant differences (***, *P* < 0.001; *, *P* < 0.05) between the same ECM fungal cultures in the absence and presence of supplemental inorganic N based on an independent samples *t*‐test. A, *Amanita rubescens*; L, *Lactarius rufus*; H, *Hebeloma velutipes*; S, *Suillus variegatus*; A/+L, monocultures and mixed cultures of *Amanita rubescens* and *Lactarius rufus*; A/L + H/S, mixed cultures of *Amanita rubescens* with *Hebeloma velutipes* and *Suillus variegatus*, as well as *Lactarius rufus* with *Hebeloma velutipes* and *Suillus variegatus*; H/+S, monocultures and mixed cultures of *Hebeloma velutipes* and *Suillus variegatus*.

We quantified changes in ECM biomass by comparing mycelial biomass after a 30‐d incubation in SOM extracts, with initial biomass measurements taken before the SOM extract was introduced. In monocultures without inorganic N, the biomass of *Hebeloma velutipes* and *Suillus variegatus* increased, whereas the biomass of *Amanita rubescens* and *Lactarius rufus* either decreased or remained largely unchanged (Fig. 1a). Following the addition of inorganic N, mycelial biomass of all ECM species increased, particularly for *Hebeloma velutipes* and *Suillus variegatus* (Fig. 1a). In mixed cultures, the *Amanita rubescens* and *Lactarius rufus* combination experienced a reduction in biomass in the absence of inorganic N (Fig. 1b). By contrast, other combinations of ECM species showed an increase in biomass, irrespective of the presence or absence of inorganic N (Fig. 1b).

The PCA of SOM extracts showed that the first two principal components explained 84.6% of the variance (PC1, 57.1% and PC2, 27.5%), with significant compositional differences observed among various ECM species cultures (Fig. 1c). The SOM Shannon diversity index values, derived from comparisons between SOM extracts in which ECM fungi were grown and controls without ECM fungi, indicated that monocultures of *Amanita rubescens* and *Lactarius rufus*, as well as their mixed cultures, resulted in SOM extracts with the lowest SOM diversity (Fig. 1d). Conversely, SOM extracts from cultures containing *Hebeloma velutipes* or *Suillus variegatus* exhibited the highest SOM diversity (Fig. 1d). Additionally, the addition of inorganic N significantly increased SOM diversity across all ECM fungal cultures compared with conditions lacking inorganic N (Fig. 1d).

### Soil organic compound decomposition by ECM species varied with environmental conditions

Response ratios of N and C compounds were obtained by comparing their relative abundance in SOM extracts which contained fungi, with those in control samples without fungi. These values indicate the extent of organic compound decomposition (Fig. S3), with lower response ratios indicating greater decomposition.

**Fig. 2:**
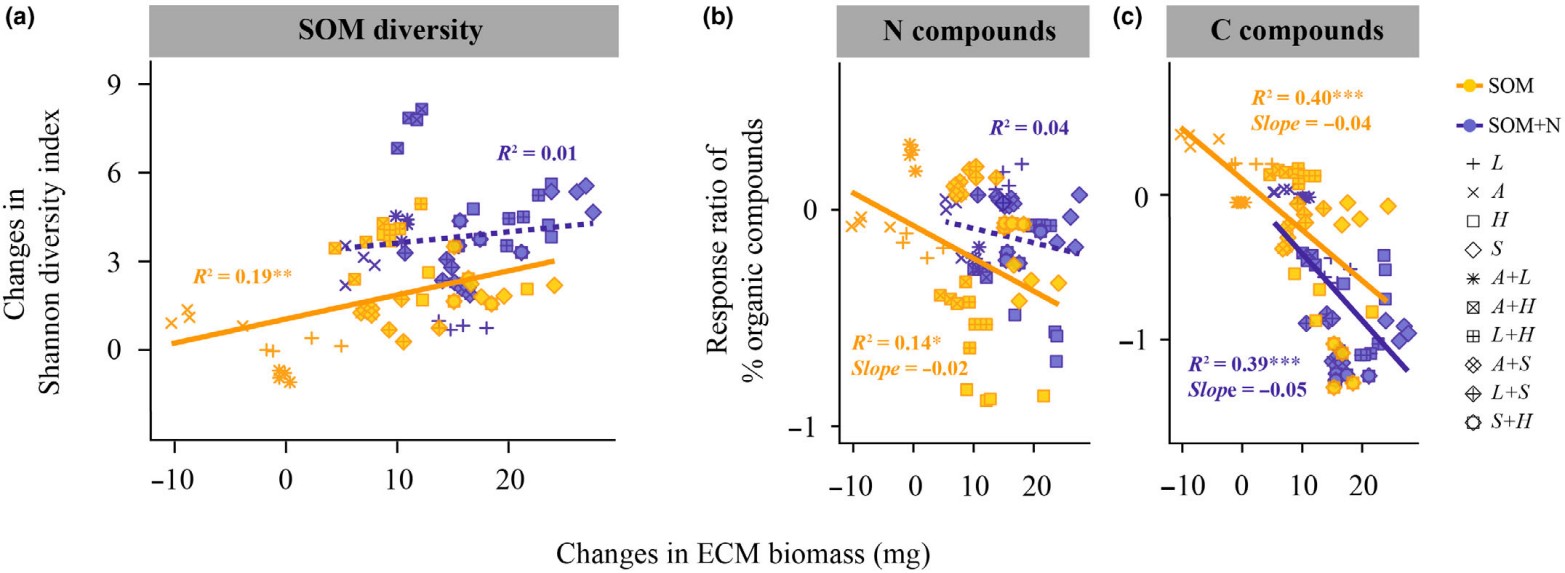
Ectomycorrhizal (ECM) fungal decomposition of soil organic matter (SOM) in response to inorganic nitrogen availability. Correlations between (a) changes in ECM biomass and the SOM Shannon diversity index, and (b, c) changes in ECM biomass and the response ratio of the percentage of nitrogen (N) and carbon (C; polysaccharide and lignin combined) compounds, determined by pyrolysis gas chromatography mass spectrometry, when inorganic N is either absent or present. ECM biomass changes were assessed by comparing mycelial biomass before and after 30 d of incubation in SOM extracts, and SOM diversity shifts were evaluated by comparing ECM‐treated SOM extracts with controls. Response ratios are calculated using the formula: (treatment‐control)/control. More negative response ratios signify increased decomposition of these compounds. The dotted and solid lines indicate statistically nonsignificant and significant (***, *P* < 0.001; **, *P* < 0.01; *, *P* < 0.05) correlations respectively, based on a linear regression estimated using ordinary least squares. A, *Amanita rubescens*; L, *Lactarius rufus*; H, *Hebeloma velutipes*; S, *Suillus variegatus*.

In the absence of inorganic N, there was a significant (*P* < 0.01) positive correlation between ECM biomass and SOM diversity (Fig. 2a). Simultaneously, ECM biomass showed a significant (*P* < 0.05) negative correlation with response ratios of N and C compounds (Fig. 2b,c). Following the introduction of inorganic N, no correlation was found between ECM biomass and either SOM diversity or the response ratio of N compounds (Fig. 2a,b). However, ECM species enhanced C compound decomposition following the addition of inorganic N, as determined by the slope of C compound response ratio when plotted against ECM biomass (Fig. 2c).

**Fig. 3:**
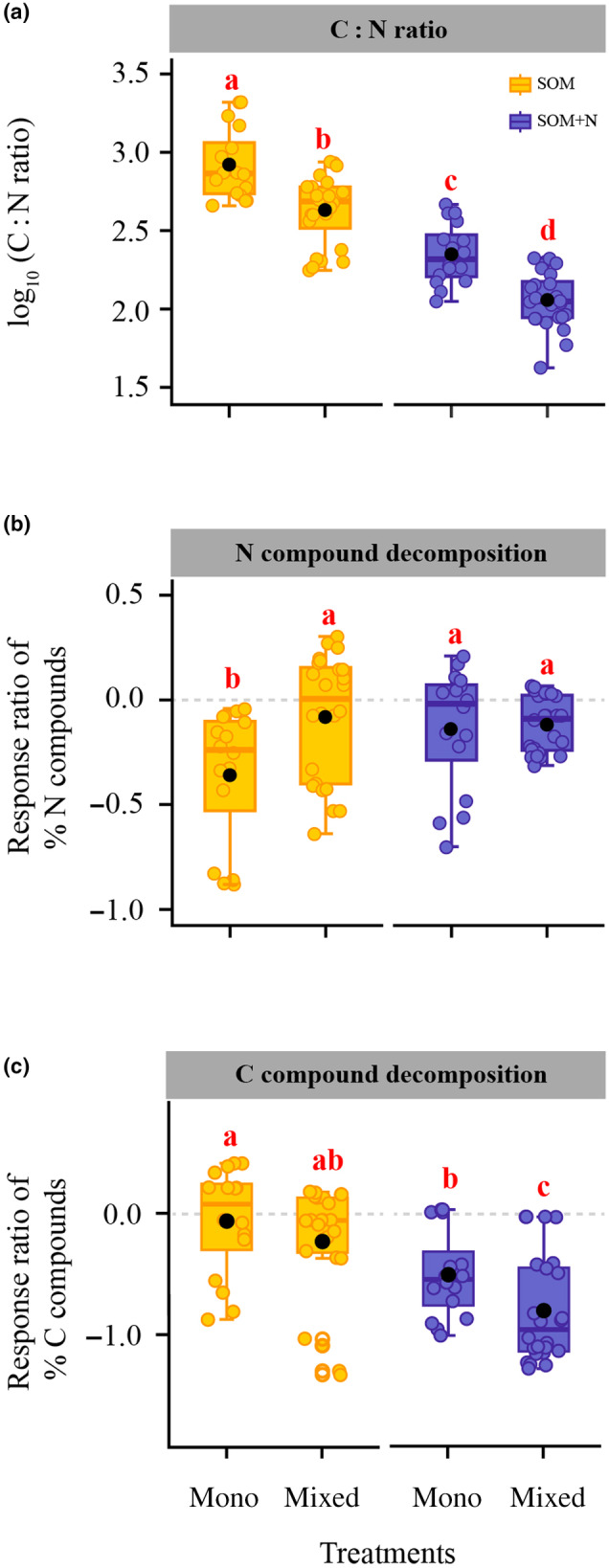
Ectomycorrhizal fungal decomposition of organic compounds in response to environmental conditions. (a) The carbon‐to‐nitrogen ratio (C : N ratio) within soil organic matter (SOM) extracts observed across different treatments. (b, c) Response ratios of the relative abundance, expressed as percentage, of nitrogen (N) and carbon (C; polysaccharide and lignin combined) compounds, determined by pyrolysis gas chromatography mass spectrometry, within SOM extracts observed across diverse treatments, calculated using the formula: (treatment‐control)/control. More negative response ratios signify increased decomposition of these compounds. Boxplots indicate medians (lines) and mean values (black points), SE and ranges, and dots are individual data points. Different lowercase letters (a, b, c and d) indicate significant differences (*P* < 0.05) based on ANOVA. mono, monocultures of four species (i.e. *Amanita rubescens*, *Lactarius rufus*, *Hebeloma velutipes*, and *Suillus variegatus*); mixed, mixed cultures of any two of the four species.

The introduction of inorganic N resulted in a reduced C : N ratio in SOM extracts, and this was observed in both monocultures and mixed cultures (Fig. 3a). Interestingly, the C : N ratio was significantly lower in mixed cultures compared with monocultures, regardless of inorganic N supplementation (Fig. 3a). The extent of N compound decomposition was greatest in monocultures lacking inorganic N, with no significant differences observed across the other three treatments (Fig. 3b). The extent of C compound decomposition was higher in mixed cultures than in monocultures, independent of the presence of inorganic N (Fig. 3c). The introduction of inorganic N further enhanced the decomposition of C compounds by ECM species in both monocultures and mixed cultures (Fig. 3c).

### ECM species decomposed recalcitrant and labile C compounds in response to environmental conditions

**Fig. 4:**
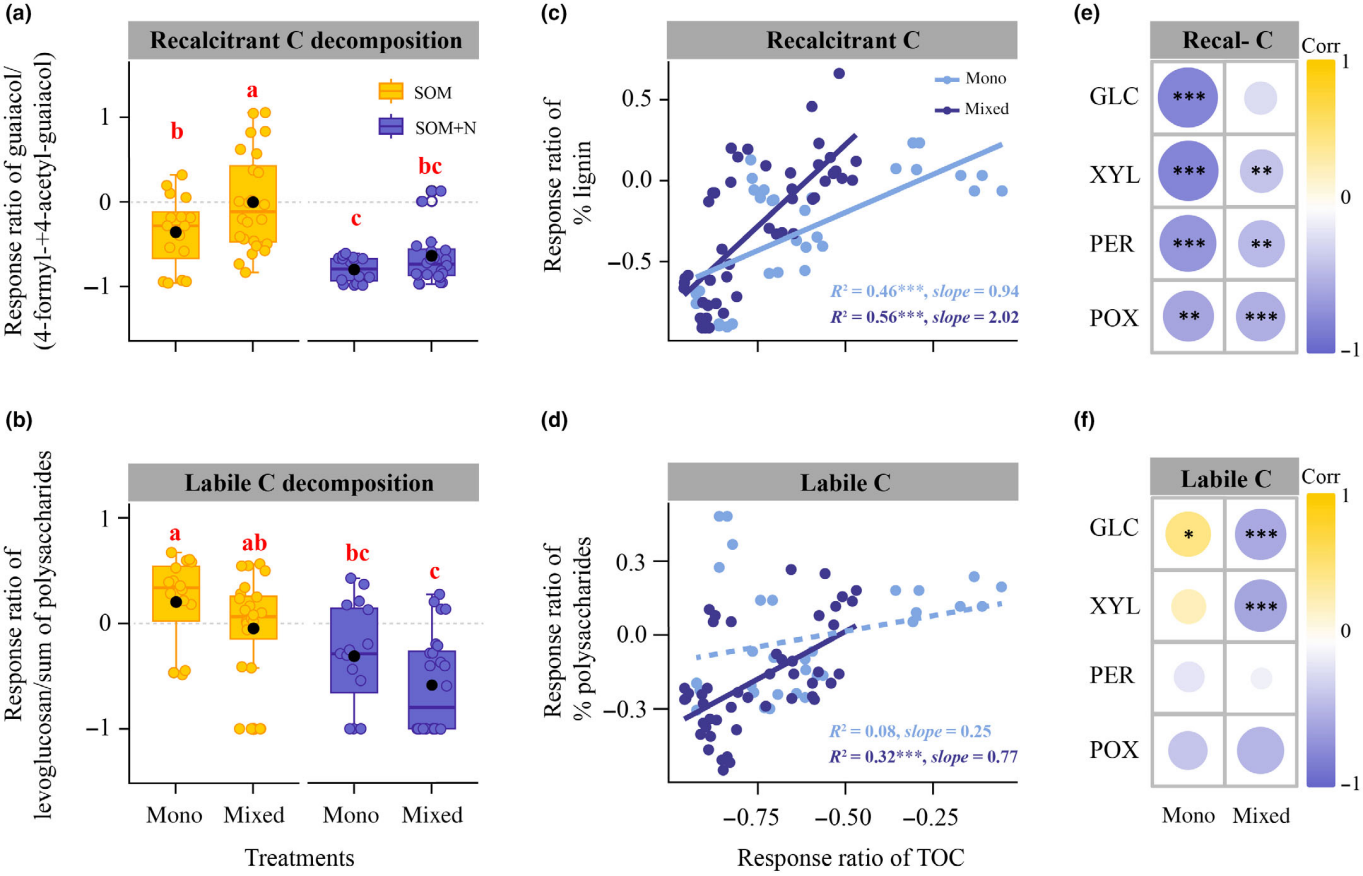
Mechanisms governing ectomycorrhizal fungal decomposition of organic carbon compounds under monoculture and mixed culture conditions. (a, b) The decomposition extent of relatively recalcitrant and labile carbon (C) compounds, as indicated by the response ratio of lignin‐derived and polysaccharide‐derived compounds (as determined by pyrolysis gas chromatography mass spectrometry), respectively, within soil organic matter (SOM) extracts across diverse treatments. Lower response ratios signify increased decomposition of these C compounds. Boxplots indicate medians (lines) and mean values (black points), SE and ranges, and dots are individual data points. Different lowercase letters (a, b and c) indicate significant differences (*P* < 0.05) based on ANOVA. (c, d) Correlations between the response ratio of total organic carbon (TOC) concentrations and that of lignin and polysaccharides within monocultures and mixed cultures. The dotted line and the solid line indicate statistically nonsignificant and significant (***, *P* < 0.001) correlations respectively, based on a linear regression estimated using ordinary least squares. (e, f) Pearson correlations between response ratios of recalcitrant (i.e. lignin) and labile C (i.e. polysaccharides) compounds and those of enzyme activities under monocultures and mixed cultures. The significance of the correlations is evaluated at the 0.05 level (***, *P* < 0.001; **, *P* < 0.01; *, *P* < 0.05). Data for the above analyses were calculated using the formula: (treatment‐control)/control. mono, monocultures of four species (i.e. *Amanita rubescens*, *Lactarius rufus*, *Hebeloma velutipes*, and *Suillus variegatus*); mixed, mixed cultures of any two of the four species; GLC, β‐glucosidase; XYL, β‐xylosidase; PER, peroxidase; POX, phenol oxidase.

The addition of inorganic N facilitated the decomposition of both recalcitrant and labile C compounds, as observed in both monocultures and mixed cultures (Fig. 4a,b). Notably, in mixed cultures, there was a decrease in the extent of recalcitrant C compound decomposition compared with monocultures, while the extent of decomposition of labile C compounds significantly increased (Fig. 4a,b). These trends were evidenced by changes in the ratios of lignin‐derived and polysaccharide‐derived compounds in SOM extracts containing ECM species relative to controls, with lower values indicating more pronounced decomposition of these C compounds (Schellekens *et al*., 2009).

There were significant (*P* < 0.001) positive correlations between the response ratio of TOC and that of lignin in both monocultures and mixed cultures, with stronger correlations in mixed cultures (Fig. 4c). No significant correlation was found between the response ratio of TOC and that of polysaccharides in monocultures, while a significant (*P* < 0.001) positive correlation was evident in mixed cultures (Fig. 4d). Additionally, in monocultures, significant (*P* < 0.05) negative correlations were observed between the response ratio of lignin and that of four enzyme activities (Fig. 4e), while no significant negative correlations were found between the response ratio of polysaccharides and that of these enzyme activities (Fig. 4f). By contrast, in mixed cultures, significant (*P* < 0.05) negative correlations were noted between the response ratio of lignin and that of XYL, PER, and POX activities (Fig. 4e), as well as between the response ratio of polysaccharides and that of GLC and XYL activities (Fig. 4f).

### Factors controlling SOM dynamics

**Fig. 5:**
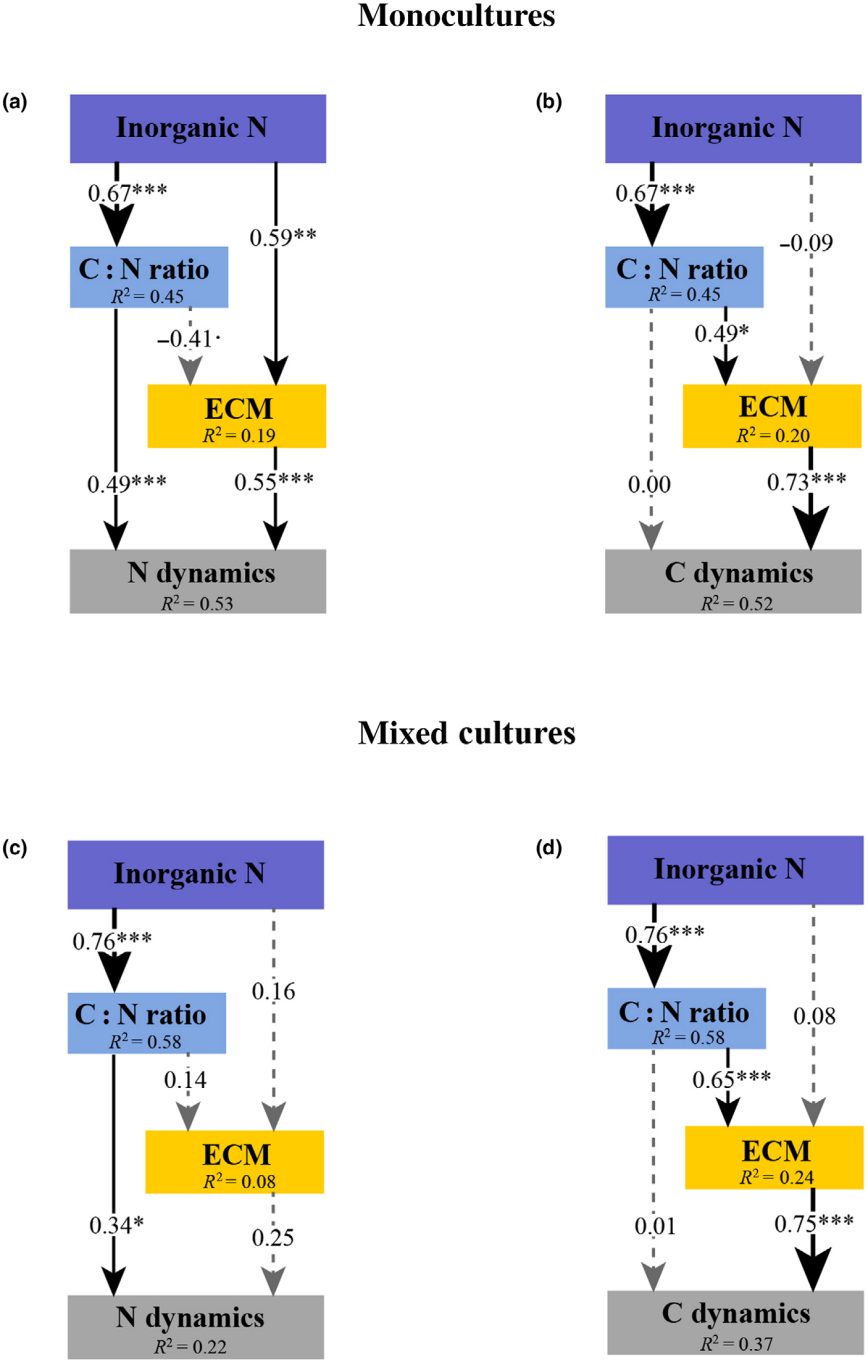
Structural equation models of soil organic matter dynamics controlled by ectomycorrhizal (ECM) fungi under varied environmental conditions. The interactions between inorganic nitrogen (N) availability, carbon‐to‐nitrogen (C : N) ratio, and the role of ECM fungi (integrating ECM biomass and enzyme activities), in influencing soil N (a, c) and C (b, d) dynamics under both monocultures (a, b) of four species (i.e. *Amanita rubescens*, *Lactarius rufus*, *Hebeloma velutipes*, and *Suillus variegatus*) and mixed cultures (c, d) of any two of the four species using structural equation models (SEMs). Soil N and C dynamics are estimate using an averaging approach that considers their content (%), composition (the first two PCA axes), and decomposition extent (ratios of N‐derived and C‐derived compounds). The *R* ^2^ denotes the proportion of variance explained for endogenous variables. Dashed and solid arrows indicate statistically nonsignificant and significant (***, *P* < 0.001; **, *P* < 0.01; *, *P* < 0.05) relationships, respectively. Arrow widths and accompanying numbers are the relative effects (i.e. standardized path coefficients) of modelled relationships. More details on the model fit are summarized in Supporting Information Table S3.

The SEMs demonstrated that the addition of inorganic N significantly (*P* < 0.001) impacted the C : N ratio within SOM extracts (Fig. 5). Interestingly, the C:N ratio did not significantly correlate with ECM fungi in explaining the N dynamics models (Fig. 5a,c). ECM fungi exhibited a significant (*P* < 0.001) positive correlation with N dynamics only in monocultures (Fig. 5a), but not in mixed culture conditions (Fig. 5c). Furthermore, SEMs describing N dynamics revealed a significant (*P* < 0.01) positive correlation between inorganic N and ECM fungi in monocultures (Fig. 5a), but no such correlation was observed in mixed cultures (Fig. 5c). The C : N ratio had a direct effect on N dynamics in both monocultures and mixed cultures (Fig. 5a,c).

In the SEMs describing C dynamics, significant (*P* < 0.05) positive correlations were observed between the C : N ratio and ECM fungi (Fig. 5b,d). Additionally, a significant (*P* < 0.001) positive correlation was observed between ECM fungi and C dynamics in both monoculture and mixed culture conditions (Fig. 5b,d). The correlation between ECM fungi and C dynamics was stronger in mixed cultures compared with that in monocultures (Fig. 5b,d). Contrary to SEMs used to elucidate N dynamics, the C : N ratio did not have significant direct effects in explaining C dynamics (Fig. 5b,d).

## Discussion

### Variability in SOM decomposition capabilities among ECM species

Our results reveal significant variations in the ability of different ECM fungal species to decompose complex and ecologically realistic SOM extracts from a Scots pine forest. Overall, *Hebeloma velutipes* and *Suillus variegatus* demonstrated significant ability to decay SOM. By contrast, *Amanita rubescens* and *Lactarius rufus* showed a distinct preference for ammonia‐N utilization (Fig. S4), coupled with a limited capability for SOM decomposition. This is inherently linked to the morphological characteristics and evolutionary histories of these species (Kohler *et al*., 2015; Pellitier & Zak, 2018).

To contextualize these findings, it is important to note that the soil extracts employed in our study were collected from Swedish forests characterized by abundant SOM but with little inorganic N (Fig. S5). *Hebeloma velutipes* and *Suillus variegatus* were able to grow on SOM extracts, whereas *Amanita rubescens* and *Lactarius rufus* could not persist under these conditions (Fig. 1a,b). Furthermore, our findings demonstrate that the extent of SOM extract modification was significantly greater for *Hebeloma velutipes* and *Suillus variegatus* compared with *Amanita rubescens* and *Lactarius rufus* (Fig. 1c,d). Our analysis is not able to disentangle the direct effects of the ECM fungi on SOM decomposition vs the contribution of organic matter to the growth media from hyphal exudation. ECM fungi are known to release a range of molecules as hyphal exudates in pure culture, including organic acids (Sun *et al*., 1999), notably oxalate (Johansson *et al*., 2008). However, we emphasize that the molecules that changed most in our experimental systems (Table S1) were generally more recalcitrant and complex than those measured in previous characterizations of hyphal exudates, and therefore, we suggest that the modifications in SOM composition in our study more likely arise from decomposition of organic matter.

In many pristine forest ecosystems, inorganic nutrients derived from both mineralization and atmospheric deposition are scarce, so nutrient availability may primarily be driven by changes in the concentrations of organic molecules, such as amino acids. Simple organic forms of N may modify competitive interactions because some ECM fungi can readily utilize N from amino acids (Hazard *et al*., 2017) and even take the molecules up intact (Näsholm & Persson, 2001). Modification of the C : N ratio of the growth substrate as a result of C in organic forms of N may also alter species interactions (Fransson *et al*., 2007). We predict that ECM fungi with higher SOM decomposition capacities, such as *Hebeloma velutipes* and *Suillus variegatus*, might become more dominant on the root systems of plants exposed to elevated organic N inputs, potentially displacing less efficient SOM‐decomposing ECM fungi.

### Inorganic N availability governs ECM fungal decomposition of SOM

Increased inorganic N availability contributes to restructuring of ECM fungal communities, favouring species with diminished SOM decay potential over those with greater capabilities (Pellitier & Zak, 2021; Argiroff *et al*., 2022; Mayer *et al*., 2023). A consensus among these studies is that this ecological shift is largely attributed to alterations in host plant nutritional needs, whereby rising soil inorganic N prompts plants to rely less on ‘costly’ ECM fungi for SOM decomposition and prefer ‘low‐cost’ inorganic N (Franklin *et al*., 2014; Bogar *et al*., 2019). Our study introduces a critical dimension by highlighting the dynamic interplay between the nutritional demands of ECM fungi for their own growth and the nutritional environment within their habitats (Plett *et al*., 2020).

Our findings indicate that the decomposition of SOM by ECM species facilitates their growth predominantly in the absence of inorganic N, with this benefit diminishing upon the introduction of inorganic N (Fig. 2a). This phenomenon is likely associated with the alteration in their mechanism for the decomposition of organic compounds subsequent to the introduction of inorganic N. For example, in pure culture conditions, we observed a correlation between increased ECM fungal biomass and N compound decomposition without inorganic N supplementation; however, this relationship was absent when inorganic N was added (Fig. 2b). This observation highlights that inorganic N addition alleviates N limitation in ECM fungi themselves (Högberg *et al*., 2021), which may be a critical factor contributing to the reduction in SOM decomposition following fertilization. This finding also provides valuable insights for interpreting field‐based observations, where it has been demonstrated that, under low‐N conditions, trees allocate more C to their roots (Högberg *et al*., 2010; Corrêa *et al*., 2011) but this does not appear to correspond to an increase in N gain from the fungus (Corrêa *et al*., 2011; Näsholm *et al*., 2013).

Soil microorganisms, including ECM fungi, are considered to be C‐limited (Zak *et al*., 2019). We found that in the absence of plant photosynthesis‐derived C supply, ECM species, particularly *Hebeloma velutipes* and *Suillus variegatus*, have the capacity to decompose organic C compounds to sustain their growth. This is evidenced by a significant correlation observed between increased ECM biomass and the decomposition of C compounds (Fig. 2c). Notably, our results reveal that inorganic N addition enhances the decomposition of C compounds by ECM species, partly because ECM fungal growth becomes primarily limited by C availability when N supply is abundant (Högberg *et al*., 2021). To maintain the balance between C and N, ECM species expend additional energy by releasing more enzymes for the decomposition of organic C compounds (Fig. S6).

Given that the enzymatic breakdown of C compounds by ECM fungi is a resource‐intensive process, these fungi primarily depend on the photosynthates provided by their tree hosts in forest ecosystems (Zak *et al*., 2019). In comparison to species that lack the capacity to decompose SOM, those capable of SOM decomposition typically exhibit greater C demands (Fig. S7), largely due to their extensive mycelial production (Clemmensen *et al*., 2015) or greater production of extracellular enzymes. The shifts in dominance from ECM taxa typified by *Suillus* or *Hebeloma* to those represented by *Amanita* or *Lactarius*, as observed in the transition from N‐poor to N‐rich soil, may offer insights into field observations indicating that limited available inorganic N in soils is associated with increased plant investment of C into ECM fungi (Hasselquist *et al*., 2012), as well as ECM fungi tending to produce less aboveground and belowground biomass in N‐polluted forests (Ekblad *et al*., 2016; Lilleskov *et al*., 2019).

### Interspecific interactions regulate ECM fungal decomposition of SOM

In natural ecosystems, individual trees are typically colonized by a diverse array of ECM fungi, leading to complex interspecific interactions among these fungal species (Kennedy, 2010). Such interactions exert a significant influence on the spatial distribution of ECM fungi and play a crucial role in shaping the structure and functions of their communities (Kennedy, 2010). To investigate how interspecific interactions among ECM fungi affect SOM decomposition, we established experimental conditions that simulated both independence and interaction of ECM species. Our findings demonstrate that in scenarios where two ECM fungi with differing SOM decomposition capacities are present, the species with limited SOM decomposition potential (i.e. *Amanita rubescens* and *Lactarius rufus*) are advantaged, particularly under conditions of inadequate inorganic N (Fig. 1b). This advantage is presumably attributable to the small‐molecule substances released by *Hebeloma velutipes* and *Suillus variegatus* during SOM decomposition, which serve as resources utilized by the co‐cultured *Amanita rubescens* and *Lactarius rufus* to facilitate their growth.

Additionally, our study reveals that ECM species exhibit a heightened demand for C relative to N under interactive conditions, particularly following the addition of inorganic N (Fig. 3). This C preference serves to support the extension of their hyphal networks and the generation of biomass (Bogar, 2023), enhancing their competitive advantage in colonizing soil resources and the root tips of their host plants within forest ecosystems (Kennedy, 2010). However, this elevated C demand, in the absence of photosynthetic C contributions, imposes some constraints on ECM species involved in SOM decomposition (Fig. 1b). In response, these species adapt their strategies for degrading different organic C forms (Fig. 4a–d). These adaptations can be attributed to changes in the extracellular enzymes released by these species under varying environmental conditions (Fig. 4e,f), potentially aimed at optimizing energy expenditure during the decomposition process.

### Mechanisms underlying ECM fungal control of SOM dynamics and its contextual dependence

We applied SEMs to elucidate the underlying mechanisms by which ECM fungi influence SOM dynamics in response to increasing inorganic N availability, considering both species‐independent and interactive conditions. Our analysis encompassed SOM dynamics, including N and C cycling. Overall, we found that with increased inorganic N availability, ECM fungi play a more important role in regulating C dynamics than N dynamics, particularly in interspecific interaction environments. Specifically, inorganic N addition significantly disrupts the C and N balance of ECM fungal habitats, intensifying the C limitation experienced by ECM species and escalated their demand for C (Ekblad *et al*., 2016). Consequently, ECM species release more extracellular enzymes for the decomposition of soil organic C compounds to sustain their growth (Kennedy *et al*., 2007).

While these results reflect fungal behaviour under culture conditions, they raise questions about how ECM fungi acquire and utilize C when in symbiosis with a plant. Interestingly, the ability of some ECM fungi to grow in culture without a host plant may reflect important differences in their biology. These culturable fungi may possess traits that allow them to more flexibly source C, such as a greater ability to use SOM or stored C reserves when plant‐derived photosynthates are unavailable. By contrast, unculturable fungi could be more specialized for direct symbiotic interactions, depending heavily on their host for C acquisition (Tedersoo *et al*., 2010).

In symbiotic conditions, C allocation from the plant may differ, with plants potentially favouring fungi that deliver more N for less C investment (Bogar *et al*., 2021). This effect could lead to shifts in ECM fungal biomass, species richness, and community composition in response to changes in photosynthate availability (Corrales *et al*., 2017; Averill *et al*., 2018). Such changes could alter the role of ECM fungi in SOM dynamics, especially if fungi turn to SOM as an alternative C source when plant‐derived C is limited (Talbot *et al*., 2008). Our results suggest that the utilization of SOM by ECM fungi is closely linked to both fungal identity and environmental contexts, but further research is needed to explore how these dynamics are shaped by symbiotic associations with plants and whether culturable fungi exhibit different ecological strategies compared with their unculturable counterparts. Genome sequencing and gene expression studies on an increasingly large range of ECM fungal species will help unpick relationships between genetic potential for SOM decomposition and responsiveness to environmental conditions, but again expansion to include less easily culturable species is critical.

In conclusion, our study indicates that ECM fungi significantly influence SOM dynamics, with their contributions intricately linked to environmental contexts. Increased inorganic N availability effectively alleviates N limitations in ECM fungi but exacerbates their C constraints. This is particularly notable in species with pronounced SOM degradation capabilities, leading to an acceleration in soil C cycling. Additionally, C limitation among ECM fungi is heightened in the presence of interspecific interactions. Such interactions between ECM species with varying degradation capacities confer advantages upon nondegrading species, as species proficient in SOM decomposition release small molecular organic substances during SOM degradation, particularly in the absence of inorganic N supplementation. However, this process prompts an increase in labile organic C compound decomposition by ECM species with significant SOM degradation abilities, aiding in energy conservation to a certain extent. These findings help shed light on the underlying mechanisms governing the observed correlations among ECM fungal community composition, soil C storage dynamics, and inorganic N availability.

## Competing interests

None declared.

## Author contributions

FC, DJ and QC conceived the idea. FC carried out the field trips and collected the samples. QC, BD and IS conducted the experiments in the laboratory. QC performed data analyses. QC wrote the first draft of the manuscript. QC, BD, DJ and FC finished the manuscript. All authors contributed to the intellectual development of this study.

## Supporting information


**Fig. S1** Schematic overview of pure culture experimental setup.
**Fig. S2** Percentage of soil organic compound groups.
**Fig. S3** Correlations between soil organic compound percentages and ratios.
**Fig. S4** Ammonia‐N response ratios in SOM extracts across treatments.
**Fig. S5** Concentrations of TOC, ammonia‐N and nitrate‐N in SOM extracts.
**Fig. S6** Response ratios of enzyme activities in SOM extracts across treatments.
**Fig. S7** Variation in TOC per milligram of ECM fungal biomass.
**Table S1** List of organic compounds in forest soils and their detection in soil extracts.
**Table S2** Correlations between soil indicators in forest soils and soil extracts.
**Table S3** Summary of model fit statistics for standardized structural equation models.
Please note: Wiley is not responsible for the content or functionality of any Supporting Information supplied by the authors. Any queries (other than missing material) should be directed to the *New Phytologist* Central Office.

## Data Availability

The data supporting the findings of this study are presented in the figures.
