# Supplementary material for: Environmental dependency of ectomycorrhizal fungi as soil organic matter oxidizers
Source: New Phytol. 2024 Oct 17;244(6):2536–47. doi: 10.1111/nph.20205 (PMC11579442; doi:10.1111/nph.20205)
Supplement: Supplementary file 1 — Fig. S1 Schematic overview of pure culture experimental setup. Fig. S2 Percentage of soil organic compound groups. Fig. S3 Correlations between soil organic compound percentages and ratios. Fig. S4 Ammonia‐N response ratios in SOM extracts across treatments. Fig. S5 Concentrations of TOC, ammonia‐N and nitrate‐N in SOM extracts. Fig. S6 Response ratios of enzyme activities in SOM extracts across treatments. Fig. S7 Variation in TOC per milligram of ECM fungal biomass. Table S1 List of organic compounds in forest soils and their detection in soil extracts. Table S2 Correlations between soil indicators in forest soils and soil extracts. Table S3 Summary of model fit statistics for standardized structural equation models. Please note: Wiley is not responsible for the content or functionality of any Supporting Information supplied by the authors. Any queries (other than missing material) should be directed to the New Phytologist Central Office. [file NPH-244-2536-s001.docx]

## *New Phytologist* Supporting Information

Article title: Environmental dependency of ectomycorrhizal fungi as soil organic matter oxidizers

Authors: Qiuyu Chen, Ilya Strashnov, Bart van Dongen, David Johnson, and Filipa Cox

Article acceptance date: 30 September 2024

The following Supporting Information is available for this article:

**Fig. S1** Schematic overview of pure culture experimental setup.

**Fig. S2** Percentage of soil organic compound groups.

**Fig. S3** Correlations between soil organic compound percentages and ratios.

**Fig. S4** Ammonia-N response ratios in SOM extracts across treatments.

**Fig. S5** Concentrations of TOC, ammonia-N and nitrate-N in SOM extracts.

**Fig. S6** Response ratios of enzyme activities in SOM extracts across treatments.

**Fig. S7** Variation in TOC per milligram of ECM fungal biomass.

**Table S1** List of organic compounds in forest soils and their detection in soil extracts.

**Table S2** Correlations between soil indicators in forest soils and soil extracts.

**Table S3** Summary of model fit statistics for standardized structural equation models.


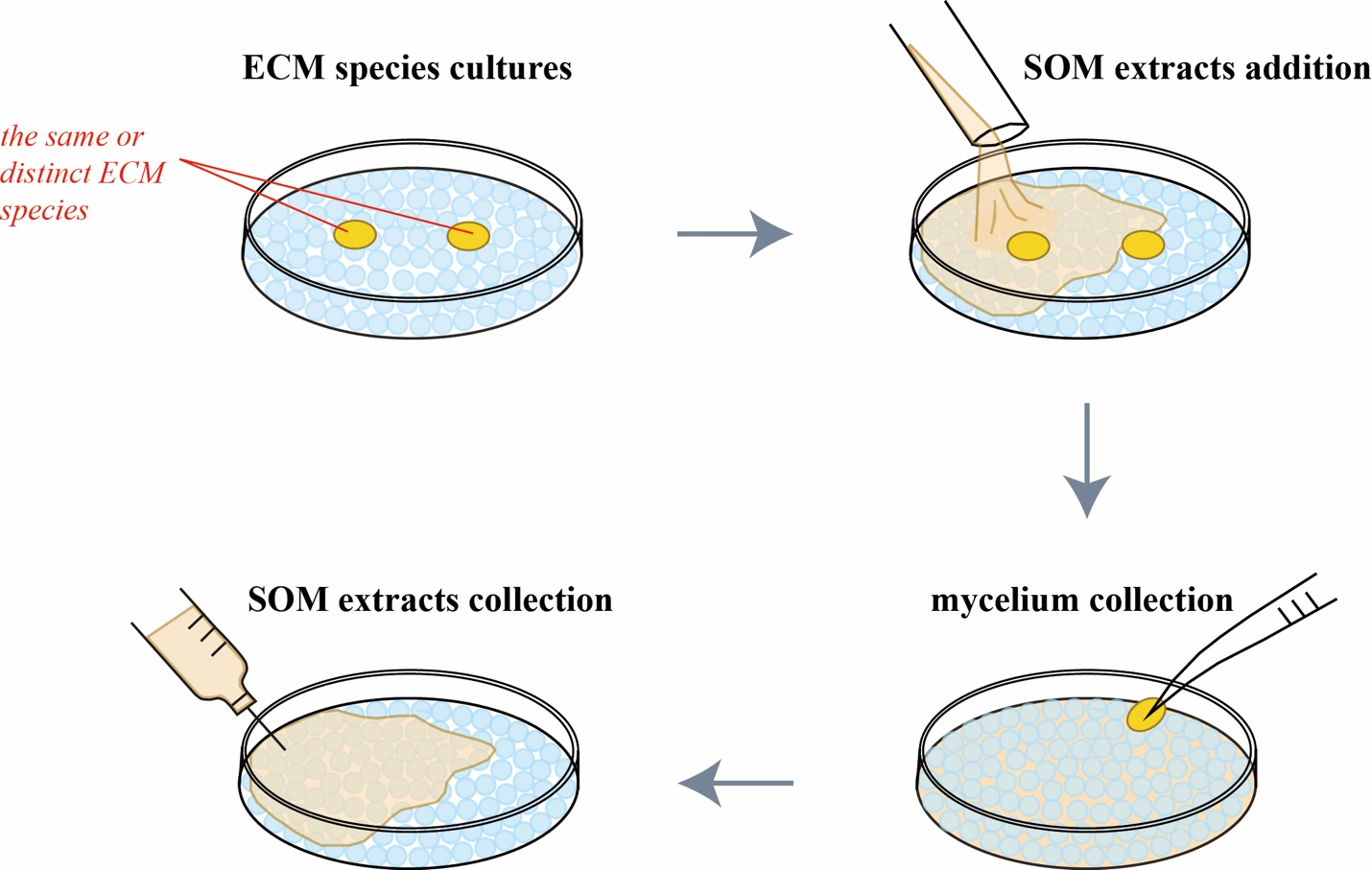


**Fig. S1** Schematic overview of ectomycorrhizal (ECM) fungal species (i.e., *Amanita rubescens*, *Lactarius rufus*, *Hebeloma velutipes*, and *Suillus variegatus*) inoculation, mycelial collection, and soil organic matter (SOM) extracts sampling.


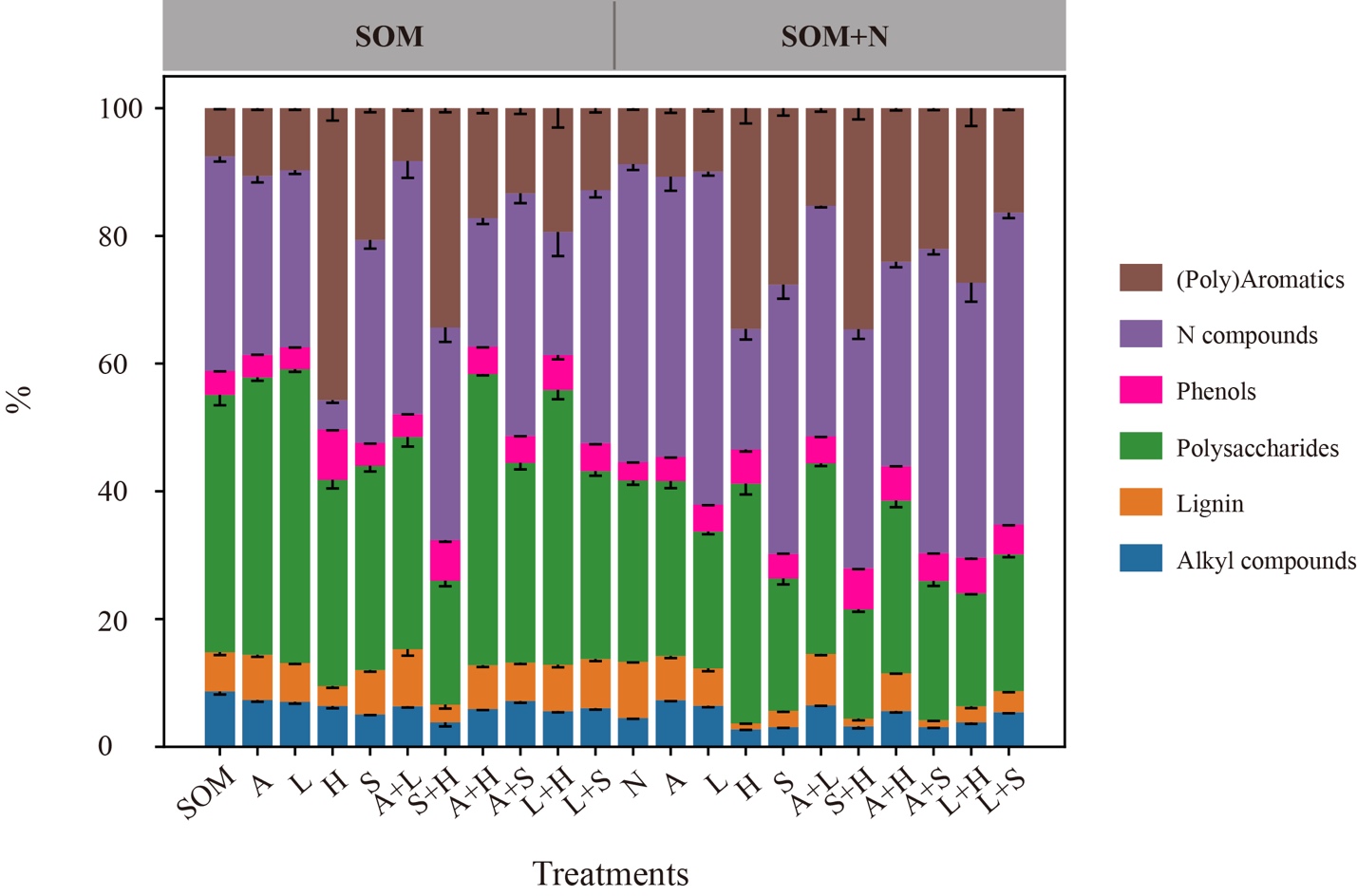


**Fig. S2** Percentage of soil organic compound groups (as defined in Table S1) identified by pyrolysis gas chromatography-mass spectrometry within soil organic matter extracts among various treatments, with data presented as mean values and standard errors (*n*=4). SOM, soil organic matter; SOM+N, soil organic matter added inorganic nitrogen; A, *Amanita rubescens*; L, *Lactarius rufus*; H, *Hebeloma velutipes*; and S, *Suillus variegatus*.

**
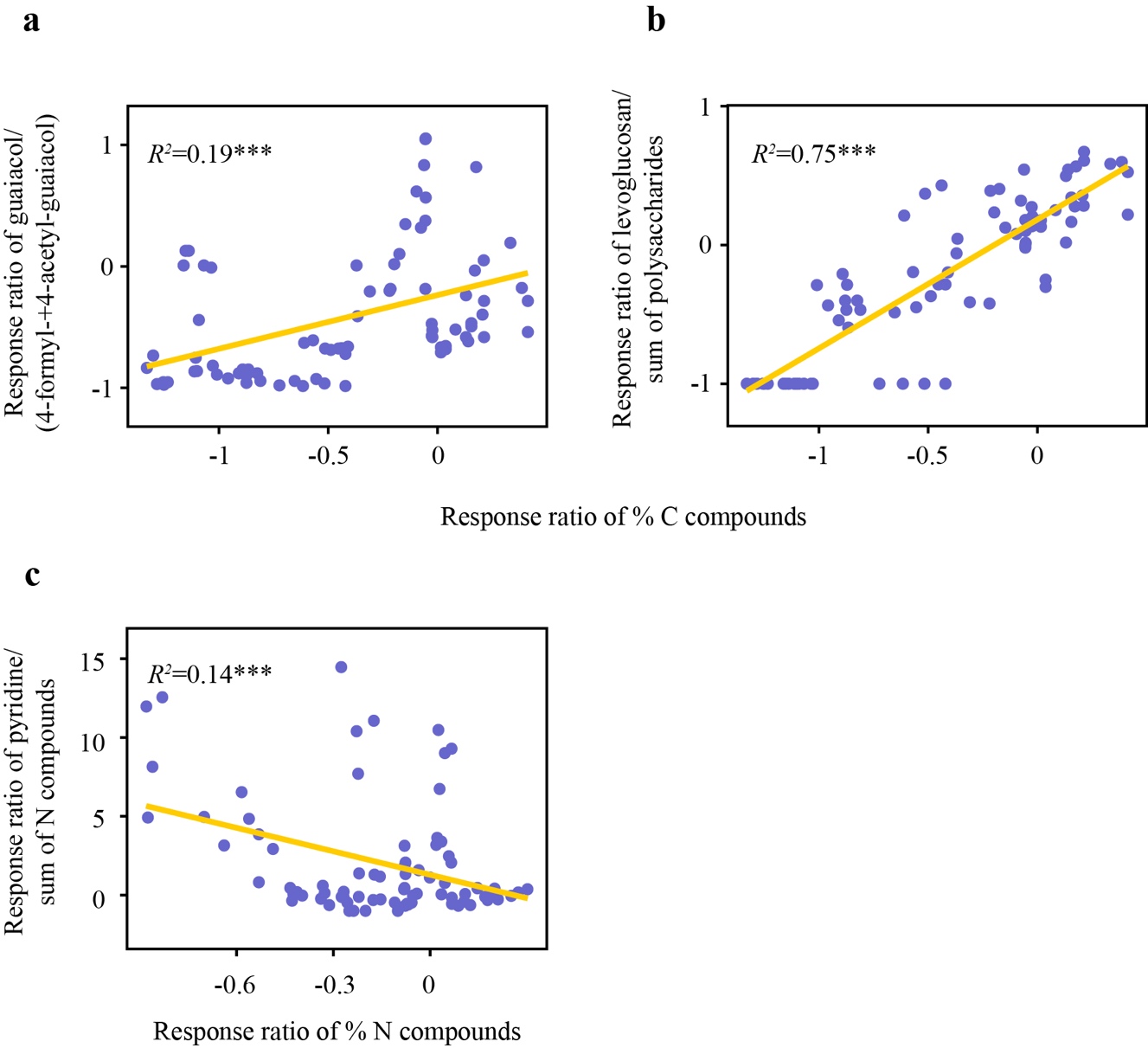
**

**Fig. S3** Correlations between the response ratio of soil organic compound percentages and that of soil organic compound ratios indicate organic compound decomposition. Decreased values of **a** guaiacol/(4-formyl-+4-acetyl-guaiacol) and **b** levoglucosan/sum of polysaccharides are indicative of enhanced decomposition of carbon (C) compounds, while an increased values of **c** pyridine/sum of nitrogen (N) compounds signals greater decomposition of N compounds. The combined relative abundance of polysaccharides and lignin is denoted as %C compounds, and the relative abundance of N-containing compounds is expressed as %N compounds. Data for the above analyses were calculated using the formula: (treatment-control)/control. The solid lines indicate statistically significant (****P*<0.001) correlations based on a linear regression estimated using ordinary least squares.

**
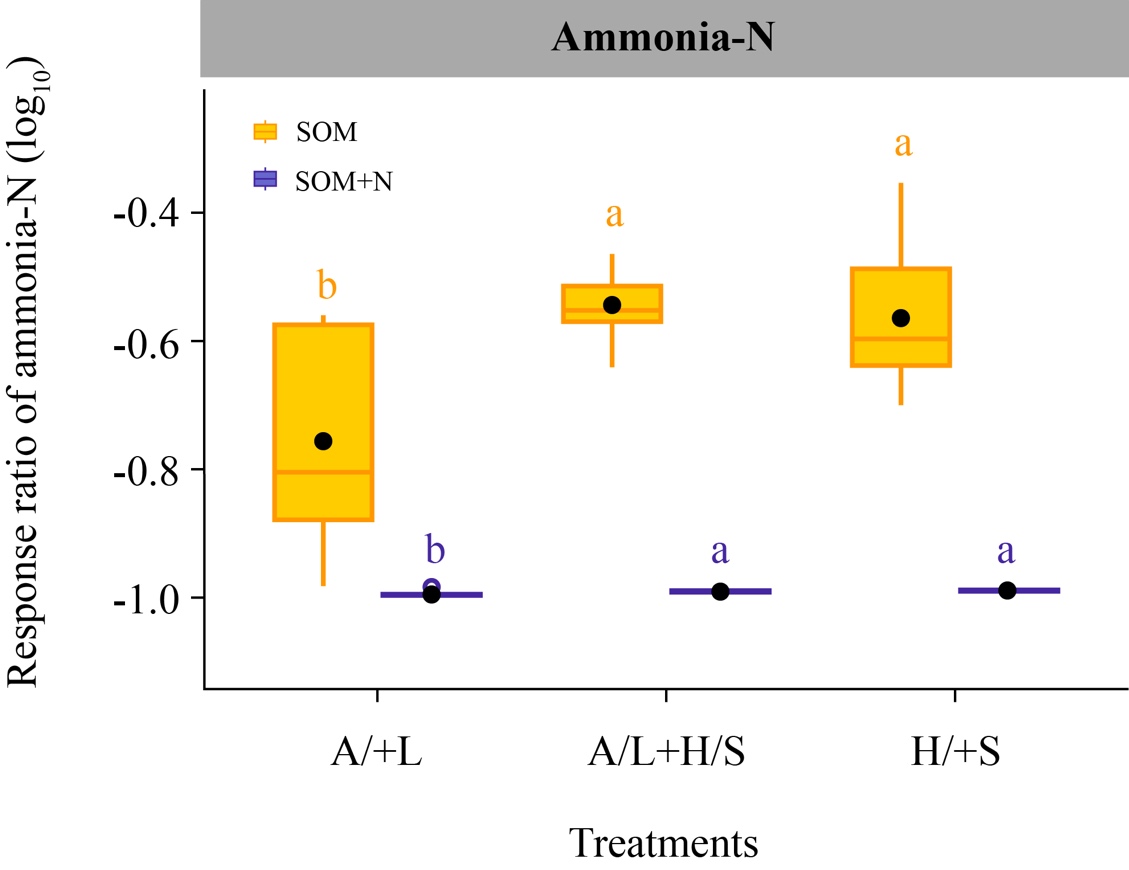
**

**Fig. S4** Response ratio of ammonia-nitrogen (N) concentrations (log_10_-transfer) within soil organic matter extracts across various treatments, calculated using the formula: (treatment-control)/control. Boxplots indicate medians (lines) and mean values (black points), and standard errors and ranges. Different yellow and purple lowercase letters (a and b) indicate significant differences (*P*<0.05) among ectomycorrhizal (ECM) fungal cultures within soil organic matter (SOM) extracts in the absence or presence of supplemental inorganic N respectively, based on ANOVA. A, *Amanita rubescens*; L, *Lactarius rufus*; H, *Hebeloma velutipes*; S, *Suillus variegatus*; A/+L, monocultures of *Amanita rubescens* and *Lactarius rufus*, and their mixed cultures; A/L+H/S, mixed cultures of *Amanita rubescens* with *Hebeloma velutipes* or *Suillus variegatus*, and *Lactarius rufus* with *Hebeloma velutipes* or *Suillus variegatus*; H/+S, monocultures of *Hebeloma velutipes* and *Suillus variegatus*, and their mixed cultures.


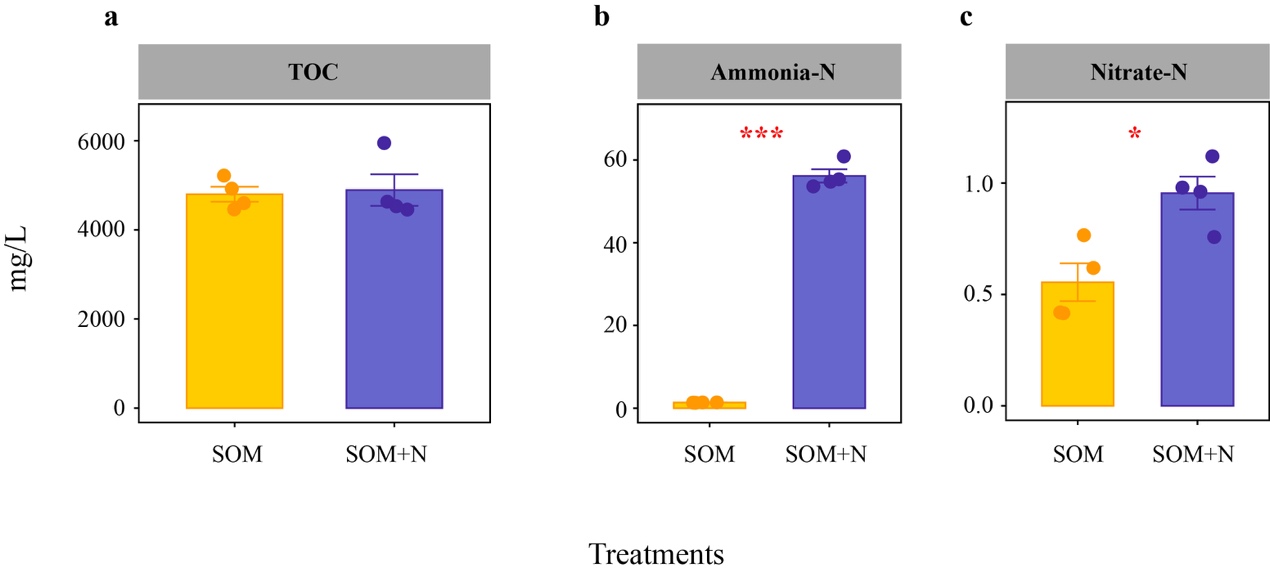


**Fig. S5** The concentrations of **a** total organic carbon (TOC), **b** ammonia-nitrogen (N) and **c** nitrate-nitrogen (N) within soil organic matter (SOM) extracts in the absence (SOM) or presence (SOM+N) of supplemental inorganic N, with data presented as mean values and standard errors (*n*=4). Red asterisks represent significant differences (****P*<0.001, **P*<0.05,) based on an independent samples *t*-test.

**
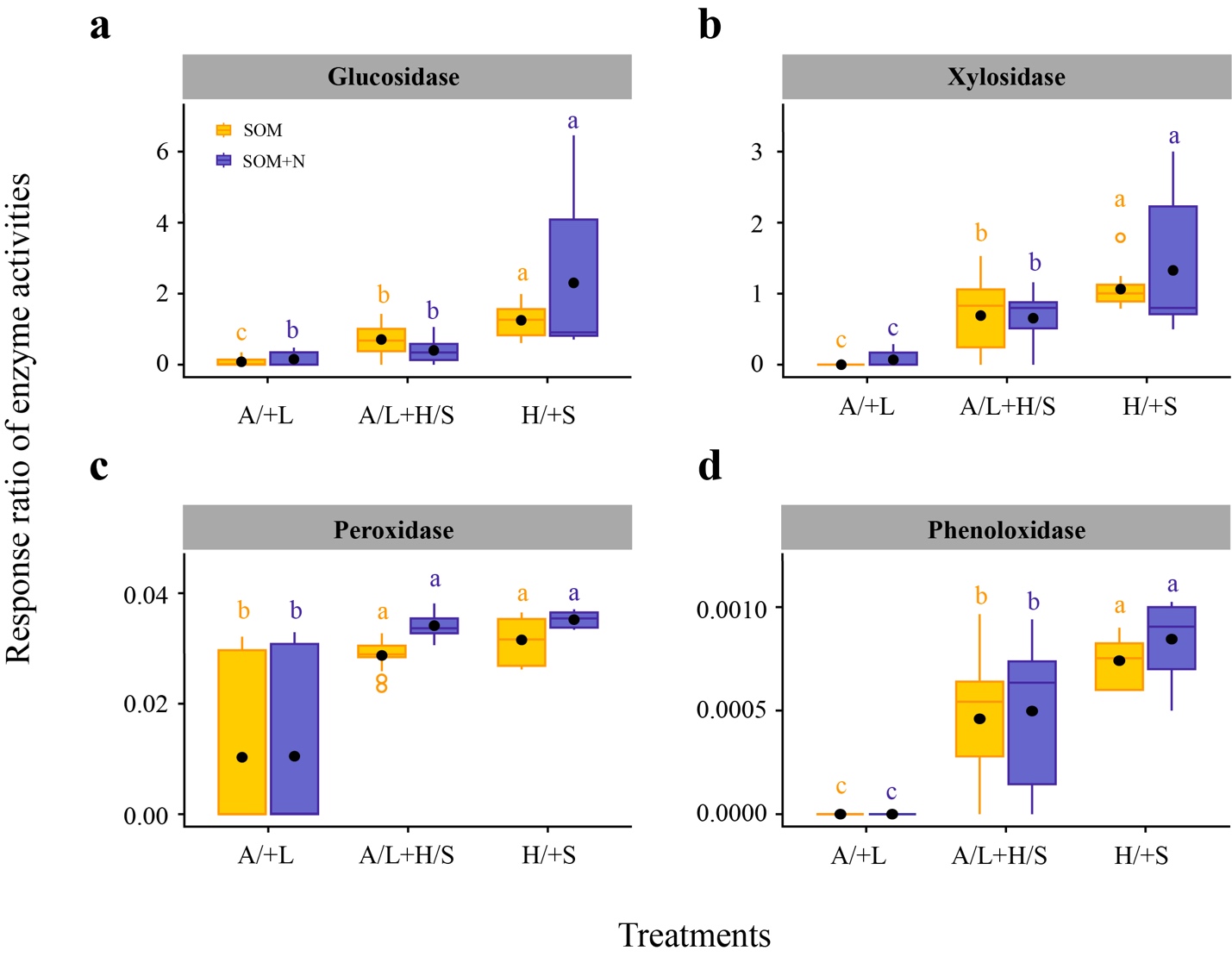
**

**Fig. S6** Response ratio of enzyme activities within soil organic matter extracts across various treatments, calculated using the formula: (treatment-control)/control. Boxplots indicate medians (lines) and mean values (black points), and standard errors and ranges. Different yellow and purple lowercase letters (a, b and c) indicate significant differences (*P*<0.05) among ectomycorrhizal (ECM) fungal cultures within soil organic matter (SOM) extracts in the absence or presence of supplemental inorganic nitrogen (N) respectively, based on ANOVA. A, *Amanita rubescens*; L, *Lactarius rufus*; H, *Hebeloma velutipes*; S, *Suillus variegatus*; A/+L, monocultures of *Amanita rubescens* and *Lactarius rufus*, and their mixed cultures; A/L+H/S, mixed cultures of *Amanita rubescens* with *Hebeloma velutipes* or *Suillus variegatus*, and *Lactarius rufus* with *Hebeloma velutipes* or *Suillus variegatus*; H/+S, monocultures of *Hebeloma velutipes* and *Suillus variegatus*, and their mixed cultures.

**
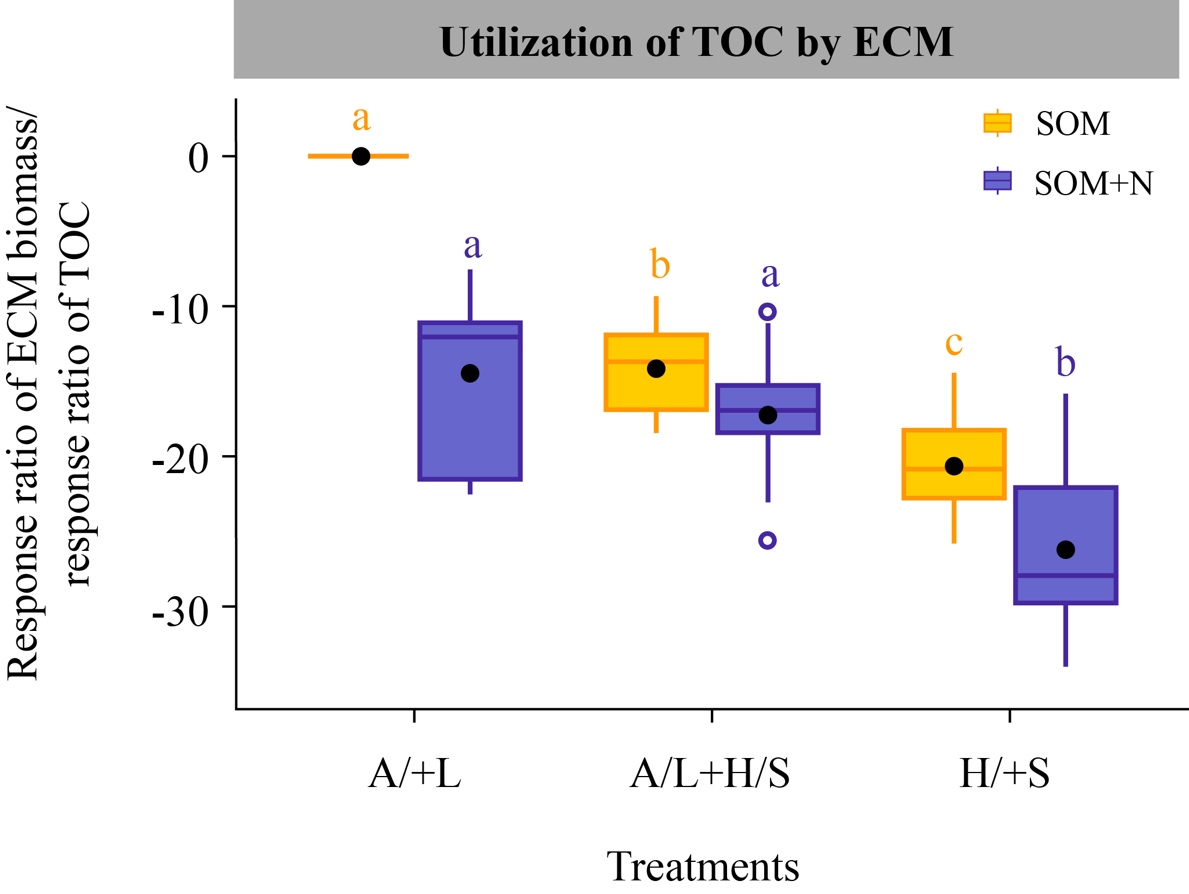
**

**Fig. S7** Variations in total organic carbon per milligram of ectomycorrhizal fungal biomass alteration. Response ratio of ectomycorrhizal (ECM) biomass and that of total organic carbon (TOC) concentrations are calculated using the formula: (treatment-control)/control. Boxplots indicate medians (lines) and mean values (black points), and standard errors and ranges. Different yellow and purple lowercase letters (a, b and c) indicate significant differences (*P*<0.05) among ECM fungal cultures within soil organic matter (SOM) extracts in the absence or presence of supplemental inorganic nitrogen (N) respectively, based on ANOVA. A, *Amanita rubescens*; L, *Lactarius rufus*; H, *Hebeloma velutipes*; S, *Suillus variegatus*; A/+L, monocultures of *Amanita rubescens* and *Lactarius rufus*, and their mixed cultures; A/L+H/S, mixed cultures of *Amanita rubescens* with *Hebeloma velutipes* or *Suillus variegatus*, and *Lactarius rufus* with *Hebeloma velutipes* or *Suillus variegatus*; H/+S, monocultures of *Hebeloma velutipes* and *Suillus variegatus*, and their mixed cultures.

**Table S1** List of organic compounds present in forest soils and detected in soil extracts using pyrolysis gas chromatography-mass spectrometry. Code: organic compound code; M+: relative molecular mass; m/z: masses used for quantification.

| Compounds in forest soils | Detected in soil extracts | Code | M+ | m/z |
| --- | --- | --- | --- | --- |
| **Alkyl compounds** |  |  |  |  |
| Alkanes, C8-C33 | Detected C8-C12 | 8:0-33:0 | 114-450 | 85 |
| Alkenes, C8-C30 | Detected C8-C11 | 8:1-30:1 | 112-434 | 83 |
| **Lignin** |  |  |  |  |
| guaiacol | Detected | Lg1 | 124 | 109+124 |
| 4-methylguaiacol | Detected | Lg2 | 138 | 123+138 |
| 4-formylguaiacol | Detected | Lg3 | 152 | 151+152 |
| 4-vinylguaiacol | Detected | Lg4 | 150 | 135+150 |
| 4-propylguaiacol | - | Lg5 | 166 | 137+166 |
| trans-propenylguaiacol | Detected | Lg6 | 164 | 149+164 |
| 4-acetylguaiacol | Detected | Lg7 | 166 | 151+166 |
| syringol | Detected | Lg8 | 154 | 154+139 |
| 4-methylsyringol | Detected | Lg9 | 168 | 153+168 |
| 4-vinylsyringol | - | Lg10 | 180 | 180+165 |
| **Polysaccharides** |  |  |  |  |
| 2-Methylfuran | Detected | Ps1 | 82 | 53+82 |
| furan-3-one | - | Ps2 | 84 | 54+84 |
| 2,5-Dimethylfuran | - | Ps3 | 96 | 95+96 |
| 2-furaldehyde | - | Ps4 | 96 | 95+96 |
| 3-furaldehyde | - | Ps5 | 96 | 95+96 |
| 2(3H) furanone, 5-methyl | Detected | Ps6 | 98 | 55+98 |
| 5-methyl 2-furfuraldehyde | Detected | Ps7 | 110 | 109+110 |
| 2-Hydroxy-6-methyl-3-cyclohexen-1-carboxylic acid | - | Ps8 | 114 | 58+114 |
| levoglucosenone | - | Ps9 | 126 | 126 |
| dianhydrorhamnose | Detected | Ps10 | 128 | 113+128 |
| 2-methylbenzofuran | Detected | Ps11 | 132 | 131+132 |
| 4,7-dimethylbenzofuran | Detected | Ps12 | 146 | 145+146 |
| levoglucosan | Detected | Ps13 | 162 | 60+73 |
| dianhydro-alpha-D-glucopyranose | Detected | Ps14 | 144 | 144 |
| **Phenols** |  |  |  |  |
| Phenol, 3-methyl- | Detected | Ph1 | 108 | 107+108 |
| 3,4-dimethylphenol | Detected | Ph2 | 122 | 107+122 |
| 4-ethylphenol | Detected | Ph3 | 122 | 107+122 |
| **N compounds** |  |  |  |  |
| pyrrole | - | N1 | 67 | 67 |
| pyridine | Detected | N2 | 79 | 52+79 |
| diketopiperazine compound | Detected | N3 | 194 | 70+194 |
| 1H-Indole, 4-methyl | Detected | N4 | 131 | 130+131 |
| N-Pyrrolidine, 2-butyl-1-methyl- | Detected | N5 | 141 | 141 |
| (1H)-pyrrole, dimethyl | Detected | N6 | 95 | 95 |
| Acetamide compound | Detected | N7 | 237 | 195+237 |
| **(Poly)Aromatics** |  |  |  |  |
| benzene | Detected | Ar1 | 78 | 77+78 |
| toluene | Detected | Ar2 | 92 | 91+92 |
| p-Xylene | Detected | Ar3 | 106 | 91+106 |
| styrene | Detected | Ar4 | 104 | 78+104 |
| Benzene, 1-ethyl-3-methyl- | Detected | Ar5 | 120 | 105+120 |
| Benzene, 1,3,5-trimethyl- | Detected | Ar7 | 120 | 105+120 |
| Benzene, 1,2,3-trimethyl- | - | Ar8 | 120 | 105+120 |
| 1H-Indene, 3-methyl- | Detected | Ar9 | 130 | 115+130 |
| 3-Methylindene | Detected | Ar10 | 130 | 115+130 |
| indene | Detected | Ar11 | 116 | 115+116 |
| Naphthalene | - | Ar12 | 128 | 128 |
| Naphthalene, dimethyl | Detected | Ar13 | 156 | 141+156 |
| 1H-indene, 2,3-dihydro-4-methyl | - | Ar14 | 132 | 117+132 |
| Naphtalene,1,2-dihydro-2-methyl | - | Ar15 | 144 | 129+144 |
| Tetramethyl phenanthrene | - | Ar16 | 234 | 219 + 234 |

**Table S2** Correlations between soil indicators in forest soils and soil extracts. TOC: total organic carbon; SOM: soil organic matter; C: carbon; N, nitrogen.

|  | Spearman's r | *P* value |
| --- | --- | --- |
| TOC (mg l^-1^) | 0.76 | **0.03** |
| SOM Shannon-diversity index | 0.85 | **0.01** |
| C compounds (%) | 0.78 | **0.03** |
| N compounds (%) | 0.74 | **0.05** |

**Table S3** Summary of the model fit statistics evaluated for the standardized structural equation models (SEMs). The models examined the effects of predictor variables on nitrogen (N) and carbon (C) dynamics within ectomycorrhizal (ECM) fungal monocultures of four species (i.e., *Amanita rubescens*, *Lactarius rufus*, *Hebeloma velutipes*, and *Suillus variegatus*) and mixed cultures of any two of the four species, and models were shown in Fig. 5. df: degrees of freedom; χ2: Chi-square; *P*: p-value of chi-square test; CFI: comparative fit index; SRMR: standardized root means squared residual; AIC: Akaike information criterion.

| **SEM model** | **Response** | **Paths** | **df** | ***χ2*** | ***P*** | **CFI** | **SRMR** | **AIC** |
| --- | --- | --- | --- | --- | --- | --- | --- | --- |
| N dynamics within monoculture conditions | N dynamics | Inoganic N->CN ratio | 1 | 0.991 | 0.319 | 1 | 0.025 | 186.39 |
|  |  | Inorganic N, CN ratio->ECM |  |  |  |  |  |  |
|  |  | CN ratio, ECM->N dynamics |  |  |  |  |  |  |
| N dynamics within mixed culture conditions | N dynamics | Inoganic N->CN ratio | 1 | 0.010 | 0.919 | 1 | 0.002 | 200.97 |
|  |  | Inorganic N, CN ratio->ECM |  |  |  |  |  |  |
|  |  | CN ratio, ECM->N dynamics |  |  |  |  |  |  |
| C dynamics within monoculture conditions | C dynamics | Inoganic N->CN ratio | 1 | 2.994 | 0.084 | 0.957 | 0.048 | 160.28 |
|  |  | Inorganic N, CN ratio->ECM |  |  |  |  |  |  |
|  |  | CN ratio, ECM->C dynamics |  |  |  |  |  |  |
| C dynamics within mixed culture conditions | C dynamics | Inoganic N->CN ratio | 1 | 0.032 | 0.858 | 1 | 0.003 | 282.14 |
|  |  | Inorganic N, CN ratio->ECM |  |  |  |  |  |  |
|  |  | CN ratio, ECM->C dynamics |  |  |  |  |  |  |
